# The Effect of High-Pressure Processing of Caprine Milk on the Production and Properties of Yoghurt

**DOI:** 10.3390/foods13091327

**Published:** 2024-04-26

**Authors:** Agnieszka Jankowska, Katarzyna Kiełczewska, Maria Wachowska, Aneta Dąbrowska, Krzysztof Siemianowski, Elżbieta Haponiuk, Katarzyna Stasiewicz

**Affiliations:** 1 Department of Process Engineering, Equipment and Food Biotechnology, University of Warmia and Mazury, Oczapowskiego 7, 10-719 Olsztyn, Poland; mari@uwm.edu.pl (M.W.); haponiuk@uwm.edu.pl (E.H.); katarzyna.stasiewicz@uwm.edu.pl (K.S.); 2 Department of Dairy Science and Quality Management, University of Warmia and Mazury, Oczapowskiego 7, 10-719 Olsztyn, Poland; kaka@uwm.edu.pl (K.K.); anetazj@uwm.edu.pl (A.D.); krzysztof.siemianowski@uwm.edu.pl (K.S.)

**Keywords:** high pressures, caprine milk, yoghurt, yoghurt culture, rheological properties, color parameters, sensory attributes, microstructure, texture

## Abstract

The aim of this study was to determine the suitability of HP-treated caprine milk for yoghurt production and to evaluate the effect of HP treatment on yoghurt properties. Reconstituted caprine milk was subjected to HP treatment (350 MPa/10 min/20 °C); a lactic acid starter culture (YC-X16, Chr. Hansen) was added. Milk was fermented at a temperature of 43 °C until pH 4.60. Bacterial counts, pH, color, rheological characteristics, texture, microstructure, and the sensory attributes of the yoghurt were determined after production and after seven days of storage at a temperature of 4 °C. HP treatment increased color saturation and whiteness index and induced a minor increase in milk pH. Minor differences in the acidification curve were noted. During storage, *Streptococcus thermophilus* counts were significantly higher in yoghurt from HP-treated than from untreated milk, whereas *Lactobacillus delbruecki* ssp. *bulgaricus* counts remained stable. A color analysis did not reveal differences between the experimental and control yoghurts. After storage, yoghurt made from HP-treated milk was characterized by thicker consistency and lower rheological stability than the control yoghurt. The micrographs of the yoghurts confirmed the differences in rheological parameters. Yoghurt produced from HP-treated caprine milk and stored for seven days received the highest scores in the sensory evaluation.

## 1. Introduction

Dairy products and foods made from milk derivatives play an important role in the socioeconomic development of both developed and developing countries. The global annual production of caprine milk is estimated at 19.2 million tones, and it accounts for more than 2.1% of total dairy production worldwide [1]. In many countries, caprine milk is the second most popular type of milk in dairy production after cow’s milk. In recent decades, the production of caprine milk has more than doubled, and it is expected to increase by 53% by 2030 [2].

In comparison with cow’s milk, caprine milk has a higher content of total solids, total protein, casein, fat, and minerals, which contributes to its higher nutritional value. Caprine milk is also characterized by a higher proportion of short-chain fatty acids [3,4]. Caprine milk is consumed directly or processed into various dairy products, including fermented milk beverages, cheeses, ice creams, and many others [5,6,7,8]. Caprine milk is dried due to the limited size of goat populations and seasonal variations in milk output. Milk powder has a better keeping quality, requires less storage space, and involves lower transportation costs [9].

Goaty flavor is strongly related to branched medium-chain fatty acids (3-methylbutanoic acid, 4-methyloctanoic acid, and 4-methyl octanoic acid) [10]. Yoghurt made from caprine milk also has a distinctive goaty taste due to a higher content of caproic, caprylic, and capric fatty acids relative to cow’s milk, which can negatively affect consumer acceptance [5,11]. Caprine milk yoghurt is characterized by lower firmness, thinner consistency, and higher susceptibility to syneresis than yoghurt made from cow’s milk [3,12]. Numerous attempts have been made to reduce the intensity of the characteristic taste and aroma of caprine milk yoghurt, which may be unacceptable for some consumers. Various yoghurt additives have been tested, including *Kluyveromyces marxianus* yeast strains [13], *Lactobacillus acidophilus* LA-5 [14,15], *Lactobacillus rhamnosus* GG [16], *Bifidobacterium animalis*, *Lactobacillus casei* and *Lactobacillus plantarum* [15], *Lactococcus lactis* [5], *Limosilactobacillus mucosae* CNPC007 [17], fat substitutes, inulin, maltodextrin [11,14], xique-xique flour [18], cupuassu pulp [14,19], whey protein [11,12], and milk protein isolate [20]. The addition of *Leuconostoc lactis* to a traditional yoghurt starter culture minimized undesirable properties (sour, salty, and “goaty”) and enhanced desirable attributes (sweet, creamy) in caprine milk yoghurt, thus increasing its appeal for consumers and overall consumer acceptability [5]. According to Park et al. [21], the addition of xanthan gum and locust bean improved the firmness, consistency, cohesiveness, and viscosity of yoghurt after four weeks of storage. These additives also enhanced the viability of starter culture bacteria and *Bifidobacterium* ssp. Bioactive milk peptides and lotus seeds/lily bulb powder improved the taste and textural properties of caprine milk yoghurt during storage, decreased fermentation time, increased water-holding capacity (WHC), and inhibited the post-acidification of caprine milk yoghurt during storage [22]. Caprine milk was also subjected to high-intensity thermosonication in an attempt to improve the sensory properties of the produced yoghurt. This treatment decreased the diameter of milk fat globules and improved major quality parameters by delaying syneresis, increasing viscosity, and enhancing sensory properties during storage [23,24]. Similar results were reported by Ma et al. [25], who analyzed the impact of high-pressure homogenization (150 MPa) on the quality of milk for yoghurt production.

High-pressure processing (HPP) is a non-thermal method of food preservation [7]. High-pressure (HP) treatment modifies food ingredients, proteins in particular. Depending on the applied pressure, HPP can affect the size of casein micelles, the proportion of soluble casein fractions, whey protein denaturation, and the interactions between milk proteins and milk fat globule membrane components [26,27,28,29,30].

The changes induced by HP treatment in caprine milk could affect the sensory, physicochemical, and rheological properties of the produced yoghurt. Therefore, the aim of this study was to determine the suitability of HP-treated caprine milk for yoghurt production and to evaluate the effect of HP treatment on the quality parameters of yoghurt.

## 2. Materials and Methods

### 2.1. Milk Preparation and Yoghurt Production

**Figure 1:**
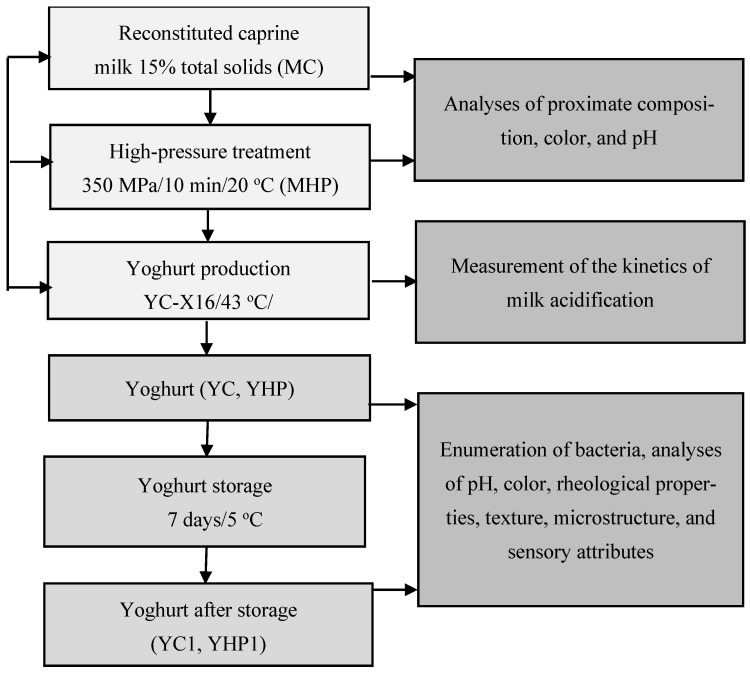
Experimental design. The following abbreviations were used to denote the analyzed samples: MC—control milk; MHP—high-pressure-treated milk; YC—yoghurt made from control milk; YHP—yoghurt made from high-pressure-treated milk; YC1—yoghurt made from control milk after storage, YHP1—yoghurt made from high-pressure-treated milk after storage.

Commercial caprine milk powder was obtained from Agro-Danmis Gramowscy (Bukowiec, Poland). Reconstituted milk (15% total solids content) was prepared by blending the appropriate amount of caprine milk powder in sterile water (121 °C/20 min). Reconstituted milk was stored overnight at 4 °C, and it was subjected to HP treatment (350 MPa for 10 min) at 20 °C in a U4040 High-Pressure Single-Chamber Apparatus (Unipress Equipment, Warsaw, Poland). The rate of adiabatic heating (~3 °C per 100 MPa) was considered during temperature measurements in the pressure holding phase (10 min). The rate of compression was 5 MPa/s, and the rate of decompression was 40 MPa/s. To produce yoghurt, untreated and HP-treated reconstituted caprine milk was heated to 43 °C, inoculated with a freeze-dried starter culture YC-X16 (Chr. Hansen, Hørsholm, Denmark), and fermented at 43 °C to pH 4.60. The produced yoghurts were stored at a temperature of 4 °C for seven days. The course of the experiment is shown in Figure 1. 

### 2.2. Chemical Composition

The proximate composition of milk, including total solids, fat, lactose, total protein, and casein content, were determined by Fourier-transform infrared (FTIR) spectroscopy in a MilkoScan™ FT1200 apparatus (Foss, Hillerøed, Denmark).

### 2.3. Kinetics of Milk Acidification

The pH of milk during acidification was measured using a multi-channel pH/pC/mV multiplexer (Cerko, Gdynia, Poland) with ERH-13-6 buried electrodes (Hydromet, Gliwice, Poland). The electrodes were immersed in milk right after starter culture addition. The pH was monitored in the Cerko Lab System program (Cerko, Gdynia, Poland). The results of pH measurements were recorded every 5 min until the pH value decreased to 4.60.

### 2.4. pH

The pH of non-fermented milks and yoghurts was determined with a CP 505 pH meter (Elmetron, Zabrze, Poland) equipped with an IJ-44C IONODE electrode and calibrated with standard solutions with pH 4.0 and 7.0 (Merck, Warsaw, Poland).

### 2.5. Microbiological Analysis

Bacteria were enumerated in yoghurt samples by the plate count method. Yoghurt samples in 1 mL aliquots were combined with 9 mL of sterile saline solution (NaCl 0.9%, *w*/*v*). Coliforms were enumerated at 30 °C on Violet Red Bile Glucose agar (VRBG) (Merck, Warsaw, Poland). Starter cultures were enumerated on selective media based according to IDF standards [31]. *Lactobacillus delbrueckii* ssp. *bulgaricus* strains were isolated on MRS agar (Merck, Warsaw, Poland) and adjusted to pH 5.4 by microaerophilic incubation in an anaerobic jar with Anaerocult C (Merck, Warsaw, Poland) at 37 °C for 72 h. *Streptococcus thermophilus* strains were isolated on M17 agar (Merck, Warsaw, Poland), adjusted to pH 7.2, by aerobic incubation at 37 °C for 48 h. Microbial counts were expressed as log10 of colony-forming units (cfus) per mL of yoghurt.

### 2.6. Color Analysis

Color was measured with a CM-3500d spectrophotometer (Konica Minolta, Tokyo, Japan). The color analysis was conducted in the CIE L*a*b* system with illuminant D65, 10° observer, d/8° measurement geometry, and an 8 mm aperture. Before the analysis, the spectrophotometer was calibrated using white (L* = 96.79; a* = −0.08; b* = −0.16) and black (L* = 0.02; a* = −0.03; b* = −0.01) standards. The results of measurements were registered and analyzed in the CM-S100w Spectra Magic TM NX (Ver. 2.3) program. Color parameters L*, a*, and b* were determined. The results were used to calculate chroma (C*) and the whiteness index (WI) according to the below formulas:

(1) $$C*=\sqrt{{a*}^{2}+{b*}^{2}}$$

(2) $$WI=\sqrt{\left(100-{L*}^{2}\right)+{a*}^{2}+{b*}^{2}}$$

### 2.7. Analysis of Rheological Properties

The flow curves of yoghurt samples were plotted with the use of a RheolabQC rotational rheometer (Anton Paar, Graz, Austria) and CC27 concentric cylinders for ascending and descending shear rate sweeps in the range of 1 to 1000 1/s and 1000 to 1 1/s, respectively. The measurements were conducted at a temperature of 20 ± 0.1 °C.

#### Area of the Hysteresis Loop

The area of the hysteresis loop was calculated as the difference in the area between the flow curve for the ascending shear rate sweep (F_1_) and the flow curve for the descending shear rate sweep (F_2_). The following formulas were used:

(3) $$F_{1}=\int_{1}^{1000}K_{1}\gamma^{n_{1}}d\gamma$$

(4) $$F_{2}=\int_{1}^{1000}K_{2}\gamma^{n_{2}}d\gamma$$

(5) $$∆P=F_{1}-F_{2}$$

Symbol descriptions:

∆P—area of the hysteresis loop calculated from the power model (Pa/s);
$K_{1}$—consistency index (Pa s^n^) from the Ostwald–de Waele model for an ascending shear rate sweep;
$n_{1}—$flow index (-) from the Ostwald–de Waele model for an ascending shear rate sweep;
$K_{2}—$consistency index (Pa s^n^) from the Ostwald-de Waele model for a descending shear rate sweep;
$n_{2}—$flow index (-) from the Ostwald–de Waele model for a descending shear rate sweep;
γ—shear rate (1/s).

### 2.8. Determination of WHC

The WHC of the samples was determined using a modified centrifugation method [32]. Yoghurt (20 g) was centrifuged (Centrifuge 5804 R, Eppendorf, Hamburg, Germany) at room temperature for 15 min; relative centrifuge force = 10,700 g. The supernatant was collected and weighed. Water-holding capacity was calculated as the weight of the pellet expressed as a percentage of the weight of the total sample.

### 2.9. Texture Analysis

Yoghurt texture was evaluated with a TA.XT Plus Texture Analyzer (Stable Micro Systems, Godalming, UK) during a compression test involving a 40 mm back extrusion cell (A/BE) (compression depth—30 mm; load—0.098 N; speed—1.0 mm/s). The results were used to determine yoghurt firmness (expressed as the maximum force in the positive area of the curve), consistency (expressed as the area under the compression curve based on the registered positive loads), and cohesiveness (expressed as the maximum force in the negative area of the curve).

### 2.10. Scanning Electron Microscopy

The microstructure of the yoghurt was analyzed under a Quanta 200 scanning electron microscope (FEI Company, Hillsboro, OR, USA). The samples were fixed to the stand with carbon tape, placed in the microscope chamber and immediately frozen at a temperature of −18 °C with the use of the Peltier module. The samples were examined at an accelerating voltage of 30 kV. Micrographs were taken at 200× magnification.

### 2.11. Sensory Analysis

The sensory attributes of caprine milk yoghurts were evaluated using the modified profiling method [33] on a 7-point scale, where 1 point denotes the absence of the analyzed attribute, and 7 points denotes extremely high intensity of the analyzed attribute. The analysis was conducted in a sensory laboratory by a panel of eight assessors trained to evaluate dairy products and whose sensory sensitivity had been validated according to EN ISO 8586:2014–03 [34]. The panelists assessed the intensity of 19 sensory attributes and the overall acceptability of the examined yoghurts. Yoghurt samples were coded, and the sensory analysis was conducted after production and storage.

### 2.12. Statistical Analysis

The results of chemical composition, pH, microbiological, color, texture, and sensory analyses were processed by one-way analysis of variance (ANOVA) and Fisher’s LSD test. All results were processed in Statistica 13.3 PL software (TIBCO Software Inc., Tulsa, OK, USA) at a significance level of 0.05. Coefficients of determination were calculated for the rheological parameters of the Ostwald–de Waele model.

## 3. Results and Discussion

### 3.1. Milk Characteristics and Acidification Profile

High-pressure treatment did not induce statistically significant changes in milk composition (Table 1). Milk pH was 6.52 (±0.03) and increased after pressurization to pH 6.56 (±0.02), which could be attributed to changes in the proportion of mineral salts in HP-treated milk [35].

**Figure 2:**
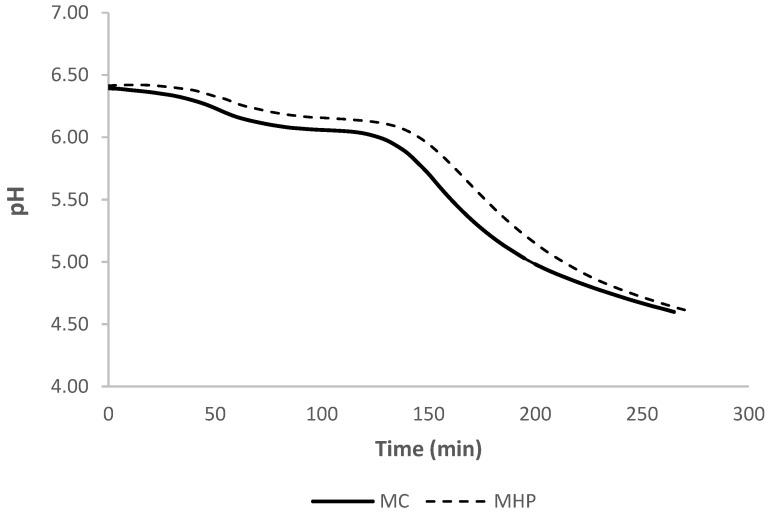
Acidification curve of control (MC) and HP-treated (MHP) reconstituted milk during fermentation with a yoghurt starter culture (YC-X16) at a temperature of 43 °C (*n* = 2).

The acidification profiles of control milk (MC) and HP-treated milk (MHP) during yoghurt production are presented by the acidification curves in Figure 2.

According to the producer’s specification of YC-X16 starter culture, 0.2 U of the starter culture per L is sufficient to bring milk (9.5% total solids) to a pH of 4.60 at a temperature of 43 °C within approximately 350 min [36], but the target pH is achieved faster in caprine milk than in cow’s milk due to lower casein content and the presence of less phosphorylated β-casein [36]. According to Park [37] and Mituniewicz-Małek et al. [38], caprine milk is acidified more rapidly due to a higher content of non-protein nitrogen and lower buffering capacity. In milk acidified with the YC-X16 starter culture, the rate of acidification is determined by *Streptococcus thermophilus* strains [36].

A comparison of the obtained acidification curves indicates that HP treatment had no negative influence on the acidification profile of caprine milk. The acidification rate of control milk did not exceed 0.05 pH/5 min in the first 135 min of the process, after which it clearly increased to a maximum value of 0.10 pH/5 min at 155–160 min, and then gradually decreased. The acidification rate of HP-treated milk did not exceed 0.05 pH/5 min in the first 145 min of the process, after which it increased to a maximum value of 0.09 pH/5 min at 165–180 min, and then decreased. A pH of 4.60 was achieved after 265 min of acidification in control milk and after 270 min in HP-treated milk.

### 3.2. pH and Microbial Counts

The pH of yoghurt produced from HP-treated milk did not differ significantly (*p* > 0.05) from that of yoghurt made from untreated milk, both after production and storage (Table 2). The acidity of both yoghurts increased significantly after storage (*p* < 0.05).

The yoghurt starter culture was composed of *Streptococcus thermophilus* and *Lactobacillus delbrueckii* ssp. *bulgaricus* symbiotic strains, which provide yoghurt with desirable properties such as pH, taste, aroma, and consistency [39]. Changes in yoghurt pH are influenced by the composition and viability of the starter culture, milk composition, and storage conditions. *Lactobacillus delbrueckii* ssp. *bulgaricus* strains are responsible for the increase in acidity after fermentation. Post-fermentation changes during storage affect the sensory attributes of yoghurt, mainly taste and aroma. The proportion of rod-shaped bacteria should be controlled to prevent excessive acidity [40,41,42].

The counts of *Streptococcus thermophilus* and *Lactobacillus delbrueckii* ssp. *bulgaricus* after production and after seven days of storage at 5 °C are presented in Table 2. *Streptococcus thermophilus* counts exceeded *Lactobacillus delbrueckii* ssp. *bulgaricus* counts in all yoghurt samples. Directly after production, *Streptococcus thermophilus* counts were higher in yoghurt made from HP-treated than untreated milk, but the difference was not significant (*p* > 0.05). In YC1, YHP, and YHP1 yoghurts, *Streptococcus thermophilus* counts were significantly higher than the counts of *Lactobacillus delbrueckii* ssp. *bulgaricus* (*p* < 0.05). A higher increase in *Streptococcus thermophilus* counts can be attributed to the higher content of amino acids in products made from pressurized milk what promotes the growth of these bacteria [43]. Coliform bacteria were not identified directly after production or after seven days of storage at a temperature of 5 °C, which indicates that yoghurts made from HP-treated and untreated reconstituted caprine milk were consistent with the microbiological quality standards for fermented milks [44].

### 3.3. Color

High-pressure treatment decreased the values of L* and, in consequence, whiteness. All samples were characterized by negative values of parameter a* and positive values of parameter b*, which points to the contribution of green and yellow components, respectively. Milk was characterized by higher saturation of greenness (a*) and yellowness (b*). As a result, the values of C* and WI increased in HP-treated milk (Table 3). Despite significant differences in the color parameters of untreated and HP-treated milk, no such differences were observed in the color parameters of yoghurt directly after production and after storage. A decrease in the L* values of milk was also reported by Gervilla et al. [45] and Stratakos et al. [46]. High-pressure treatment decreases L* values by changing the size of casein micelles [27,47] and milk fat globules [29,45]. Larger casein micelles and milk fat globules decrease light scattering [29]. Color parameters are influenced by changes in protein and fat dispersion during HP treatment. In the present study, changes in the color parameters of milk, induced by changes in milk particle dispersion, had no effect on the color parameters of yoghurt, irrespective of HP treatment or storage. High-pressure treatment did not affect the color of yoghurt in a study by Walker et al. [48].

### 3.4. Rheological Properties

Yoghurt is a non-Newtonian fluid characterized by highly complex rheological properties. Yoghurt displays time-dependent thixotropy and shear-thinning behavior [38,49]. The flow characteristics of yoghurt curd are influenced by numerous technical and technological factors. These factors are associated with a product’s composition and the production process, and they determine the physical parameters and quality of the end-product. For instance, milk pressurization strongly affects the rheological properties of yoghurt by increasing viscosity, improving structure and consistency, and preventing whey syneresis [20,50]. Shear stress values determined at different shear rates indicate that the analyzed yoghurts exhibited properties of non-Newtonian fluids. An increase in the shear rate induced non-linear changes in shear stress. Yoghurt samples were subjected to ascending and descending shear rate sweeps, and the generated hysteresis loop revealed that the rheological properties of the tested samples were unstable. The experimental data demonstrated that the analyzed samples were characterized by complex flow curves (σ = f(γ)).

**Figure 3:**
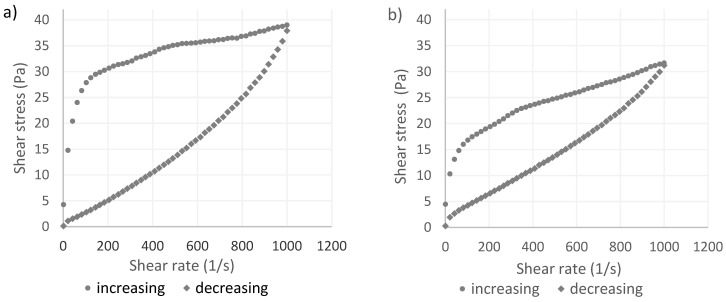

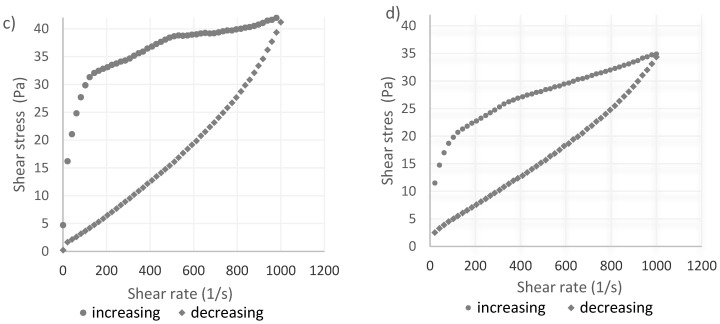
Flow curve of yoghurts: (**a**) YC—yoghurt produced from untreated reconstituted milk; (**b**) YC1—yoghurt produced from reconstituted milk after storage; (**c**) YHP—yoghurt produced from HP-treated milk; (**d**) YHP1—yoghurt produced from HP-treated milk after storage.

Based on a preliminary analysis of flow curves (Figure 3a–d), the Ostwald–de Waele rheological model was applied to describe the relationship between shear stress σ and shear rate γ. The rheological properties of the examined yoghurts described with the Ostwald–de Waele model are presented in Table 4. In all cases, the consistency index K was higher for ascending than descending shear rate sweeps, and it was determined at 4.53 to 7.29 (Pa s^n^) for ascending shear rate sweeps and at 0.18 to 0.06 (Pa s^n^) for descending sweeps. The flow index decreased from 0.25 to 0.27 for ascending shear rate sweeps and increased from 0.71 to 0.81 for descending shear rate sweeps. These results indicate that yoghurt produced from HP-treated milk had a thicker consistency because HP treatment modified the structure of milk proteins. The rheological stability (shear strength) of yoghurt was determined by comparing the area of the hysteresis loop calculated based on the shear stress values for ascending and descending shear rate sweeps. These values denote the degree of sample damage during shear rate sweeps in the range of 1 to 1000 1/s in 1200 s, while shearing of the sample at 1000 1/s lasted 120 s. The results indicate that YC and YHP were characterized by higher rheological stability than YC1 and YHP1. The area of the hysteresis loop was largest for YHP1 (∆P = 20,841.57 Pa/s), which suggests that this yoghurt was least rheologically stable and most susceptible to structural deformation under exposure to shear stress. In turn, YHP was characterized by the smallest area of the hysteresis loop (∆P = 11,393.72 Pa/s) and the greatest rheological stability.

Thixotropy usually occurs in systems that tend to regenerate their structure, including in yoghurt. The presence of a hysteresis loop during flow curve measurements at ascending and descending shear rate sweeps is indicative of thixotropy. All of the tested yoghurts exhibited thixotropic properties under exposure to varied shear rates. A high value of the coefficient of determination (R^2^ = 0.99) indicates a good fit between the data and the empirical flow curve. Apparent viscosity changed during shear testing, and it was always lower at descending than ascending shear rate sweeps.

### 3.5. Textural Properties

The WHC of yoghurt prepared from HP-treated reconstituted milk (YHP) was higher than the WHC of control yoghurt after production (Table 5); the difference was statistically significant (*p* < 0.05). After one week of storage, WHC was also higher in yoghurt made from HP-treated than from untreated reconstituted milk. According to Harte et al. [51], the higher WHC of yoghurt produced from pressurized milk compared to control milk can be attributed to the partial damage to casein micelles. HP treatment increased the WHC of samples and decreased the syneresis value, which suggests that pressurization induced changes related to whey protein denaturation, protein–protein interactions, and fat–protein interactions, all of which enhanced particle interactions and led to the formation of a stable gel network that strongly retained water [52]. In addition, pressurization increases the surface area of milk fat globules, which can promote interactions between proteins and fat globules, thus increasing WHC, minimizing syneresis [53], and improving the rheological properties of yoghurt. The increase in the WHC of stored yoghurt corresponds with the results reported by Domagala [54] and Gursel et al. [55], who observed a decrease in syneresis towards the end of the storage period, probably due to an increase in the WHC of the gel matrix during storage resulting in increased binding of water by milk proteins.

The values of all textural parameters were higher in yoghurt made from HP-treated than from untreated milk. In yoghurt produced from HP-treated milk, firmness and consistency increased, whereas cohesiveness decreased after storage (Table 5). These results indicate that HP treatment improved the textural properties of the yoghurt by contributing to thicker curd consistency and decreasing stickiness and mouth coating, which are regarded as undesirable traits. The textural parameters of yoghurt made from untreated milk did not change during storage.

Caprine milk yoghurts have a thinner consistency than yoghurts produced from the milk of other mammals, even when their content of total solids is high [54,56]. The above can be probably attributed to the fact that caprine milk has a low content of αs_1_-casein, which plays an important role in gel formation [57]. Caprine milk forms weak gels, and HP treatment can be applied to improve the water-binding capacity of solids, in particular proteins, and to promote the formation of thicker gels. High-pressure treatment modifies the structure of milk proteins and induces changes in the texture of yoghurt. High-pressure treatment changes the size of casein micelles and leads to the dissociation of some casein fractions that participate in the reconstruction of casein micelles in pressurized milk when the equilibrium between soluble and colloidal casein is restored [27,47]. The structural changes induced by HP treatment in whey proteins, including denaturation (mainly β-lactoglobulin), and the formation of whey protein and casein aggregates affect the functional properties of whey proteins, including their ability to stabilize emulsions, bind water, and form gels [28].

### 3.6. Microstructure

**Figure 4:**
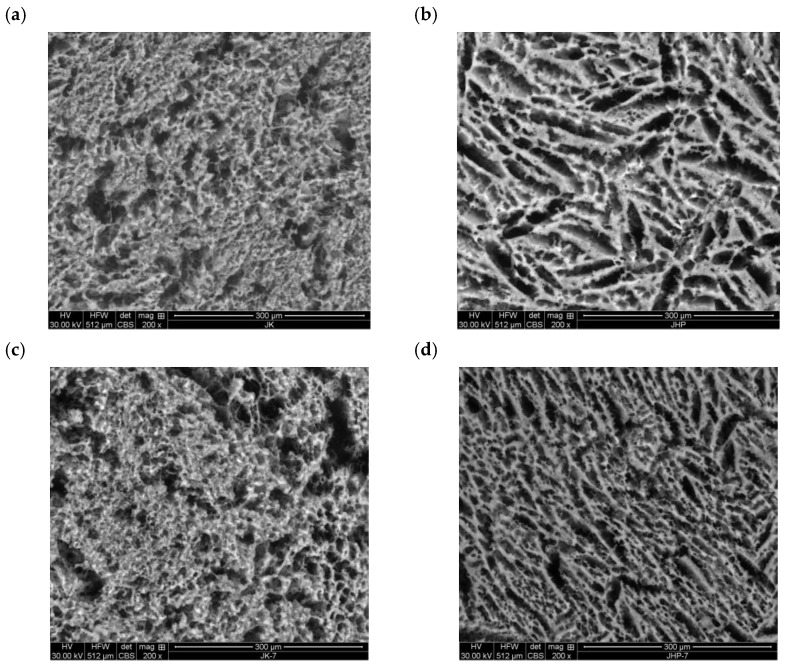
Micrographs of (**a**) yoghurt produced from untreated reconstituted milk (YC), (**b**) yoghurt produced from HP-treated reconstituted milk (YHP), (**c**) yoghurt produced from untreated reconstituted milk after storage (YC1), and (**d**) yoghurt produced from HP-treated reconstituted milk after storage (YHP1).

The micrographs of the analyzed yoghurts confirmed the observed differences in the rheological parameters of yoghurts made from untreated and HP-treated reconstituted caprine milk. The micrographs revealed that yoghurt produced from untreated caprine milk was characterized by rough and irregular curd surface, with void spaces of varied shape and size. In yoghurt made from HP-treated milk, the curd surface was smoother, and the size and shape of void spaces were more regular, particularly after storage (Figure 4a–d). A smoother curd surface indicates that water was evenly distributed in the sample, probably due to the higher water-binding capacity of proteins, and that it significantly affected the rheological properties of the yoghurt. In a study by Nguyen et al. [58], caprine milk yoghurt had a more porous microstructure (characteristic of the softest gel) than yoghurts made from cow’s and sheep’s milk. Based on the images acquired under a scanning electron microscope, Tsevdou et al. [59] also concluded that HP treatment improved the structural properties of yoghurt curd made from cow’s milk.

### 3.7. Sensory Analysis

The mean values of the sensory attributes of caprine milk yoghurts, including appearance, aroma, consistency, and taste, as well as the overall acceptability of the examined yoghurts are presented in Table 6. In terms of appearance, the analyzed yoghurts did not differ statistically in whey syneresis, intensity of creamy color, and overall color uniformity. All yoghurts were scored as extremely uniform in regard to the color (the mean scores approximate 7), but the intensity of creamy color was evaluated as very intense, with the highest mean value in sample YHP1. Whey syneresis was minimal in all examined yoghurts. None of the tested products exhibited the extensive whey syneresis that is typical of caprine milk yoghurts [3], which could be attributed to the modified production process by the use of pressurization. Yoghurt-like aroma was more detectable in stored products, and it was described as highly intense by the panelists. A similar relation was observed with regard to the sour aroma, which was described as very weak before storage and weak after storage. The tested yoghurts did not differ significantly in the intensity of the goaty aroma, but the mean scores for this attribute suggest that HP treatment induced a minor decrease in its intensity. Moreover, the intensity of goaty aroma was described as very weak. The applied starter culture and pressurization probably affected the concentrations of short-chain fatty acids (SCFAs) such as capric, caproic, and caprylic acids, for which the negative effect on the aroma of caprine milk yoghurt, compared with yoghurt made from buffalo, bovine, and ovine milk, was described by Al-Bedrani et al. [60]. An atypical aroma was not detected in any of the analyzed samples. In the sensory analysis, no significant differences (*p* > 0.05) in the uniformity of consistency, thickness, or smoothness were noted between the evaluated yoghurts. All products were characterized by extremely high uniformity of consistency, relatively low thickness, and extremely smooth mouthfeel (Table 6). The greatest differences were observed in ropiness, which was highest in the control sample directly after production (mean score of 3.9 on a 7-point scale) and lowest in yoghurt made from HP-treated milk after storage (mean score of 1.5). The tested products also differed in gelatinousness. It was found that yoghurts made from HP-treated milk had a more gelatinous consistency both before and after storage. Similar observations were made by Cruz et al. [61], who found that HP treatment of milk can significantly affect the texture of probiotic fermented milks by inducing changes in protein conformation. Moreover, in the work of Ma et al. [25], HP treatment improved hedonic scores for texture and the overall acceptability of caprine milk yoghurts.

Seven sensory attributes associated with taste were evaluated in the next stage of the sensory analysis. Significant differences were observed only in yoghurt-like taste (*p* = 0.035), which was least distinctive (moderate) in the control yoghurt (YC) and most distinctive (strong) in the yoghurt made from HP-treated milk after storage (YHP1). All analyzed products were characterized by a moderately sour taste, low sweetness, and a weak “goaty” aftertaste. However, an analysis of mean values indicates that the control yoghurt after storage (YC1) had a slightly sourer taste and a stronger “goaty” aftertaste than the remaining products. Bitter taste, salty taste, and foreign taste were not detected in any of the examined yoghurts.

After the sensory analysis, all yoghurts were evaluated for overall acceptability. Yoghurt made from HP-treated milk after storage (YHP1, mean score—5.8) received the highest score, whereas the control yoghurt after storage (YC1, mean score—4.5) received the lowest score for overall acceptability.

## 4. Conclusions

This study revealed that yoghurt produced from HP-treated caprine milk was characterized by desirable sensory properties, as demonstrated by the results of instrumental analyses, including textural parameters, rheological properties, and microstructure of yoghurts directly after production. HP treatment had no negative influence on the acidification profile of caprine milk. HP treatment did not reduce fermentation time. *Streptococcus thermophilus* counts were higher in yoghurt made from HP-treated milk than in yoghurt produced from untreated caprine milk. After storage, a greater decrease in pH was noted in yoghurt made from untreated than from HP-treated reconstituted milk. The tested yoghurts exhibited thixotropic properties because a hysteresis loop was observed in each measurement of flow curve parameters at increasing and decreasing shear rate sweeps. Yoghurt made from HP-treated milk after storage received the highest scores in the sensory evaluation, followed by the same yoghurt directly after production. Stored yoghurt produced from HP-treated milk was characterized by the most distinctive yoghurt-like taste and aroma. High-pressure treatment was also effective in reducing the goaty taste of the analyzed yoghurts after storage. The results of this study indicate that high pressure can be applied to process caprine milk for yoghurt production. However, further research is needed to optimize the parameters of HP treatment and stabilize the quality attributes of caprine milk yoghurt.

## Figures and Tables

**Table 1:** Chemical composition and pH of milk.

Parameter	Milk	
	MC	MHP
Total solids (%)	15.10 ± 0.01	15.09 ± 0.00
Fat (%)	4.71 ± 0.01	4.69 ± 0.00
Lactose (%)	5.71 ± 0.01	5.73 ± 0.00
Total protein (%)	4.01 ± 0.01	4.03 ± 0.00
Casein (%)	3.88 ± 0.04	3.94 ± 0.00
pH	6.52 ± 0.03	6.56 ± 0.02

Means ± standard deviation (*n* = 3); MC—control milk; MHP—high-pressure-treated milk.

**Table 2:** pH and starter culture counts of yoghurts.

Parameter	Yoghurt			
	YC	YHP	YC1	YHP1
pH	4.55 ^a^ ± 0.02	4.53 ^a^ ± 0.01	4.39 ^b^ ± 0.04	4.44 ^b^ ± 0.02
*Lactobacillus delbruecki* ssp. *bulgaricus* (log cfu/mL)	7.43 ^a^ ± 0.23	7.36 ^a^ ± 0.13	7.66 ^a^ ± 0.07	7.59 ^a^ ± 0.23
*Streptococcus thermophilus* (log cfu/mL)	7.73 ^a^ ± 0.14	8.13 ^b^ ± 0.10	8.08 ^b^ ± 0.18	8.27 ^b^ ± 0.11

Means ± standard deviation (*n* = 3); ^a, b^—mean values marked with different letters in the same row differ at *p* ≤ 0.05; YC—yoghurt produced from untreated reconstituted milk; YHP—yoghurt produced from HP-treated reconstituted milk; YC1—yoghurt produced from untreated reconstituted milk after storage; YHP1—yoghurt produced from HP-treated reconstituted milk after storage.

**Table 3:** Color parameters of milk and yoghurts.

Color Parameter	Milk		Yoghurt			
	MC	MHP	YC	YHP	YC1	YHP1
L*	84.88 ^b^ ± 0.01	83.64 ^a^ ± 0.21	86.27 ± 0.37	86.27 ± 0.38	86.15 ± 0.33	86.27 ± 0.40
a*	−2.97 ^b^ ± 0.08	−3.21 ^a^ ± 0.08	−2.79 ± 0.01	−2.81± 0.02	−2.80 ± 0.08	−2.81 ± 0.02
b*	4.93 ^a^ ± 0.13	5.14 ^b^ ± 0.01	5.67 ± 0.08	5.58 ± 0.04	5.78 ± 0.07	5.78 ± 0.02
C*	5.75 ^a^ ± 0.07	6.06 ^b^ ± 0.05	6.32 ± 0.07	6.32 ± 0.04	6.42 ± 0.10	6.42 ± 0.01
WI	6.94 ^a^ ± 0.06	7.29 ^b^ ± 0.06	7.32 ± 0.09	7.26 ± 0.01	7.42 ± 0.10	7.42 ± 0.02

Means ± standard deviation (*n* = 3); ^a, b^—mean values marked with different letters in the same row differ at *p* ≤ 0.05; MC—control milk; MHP—high-pressure-treated milk; YC—yoghurt produced from untreated reconstituted milk; YHP—yoghurt produced from HP-treated milk; YC1—yoghurt produced from reconstituted milk after storage; YHP1—yoghurt produced from HP-treated milk after storage.

**Table 4:** Rheological properties of yoghurts.

Rheological Properties		Yoghurt			
		YC	YHP	YC1	YHP1
Ascending shear rate sweeps	Consistency index K (Pa s^n^)	4.53	5.69	6.78	7.29
	Flow index n (-)	0.27	0.25	0.26	0.27
	Coefficient of determination R^2^ (-)	0.99	0.99	0.99	0.99
Descending shear rate sweeps	Consistency index K (Pa s^n^)	0.06	0.12	0.18	0.17
	Flow index n (-)	0.81	0.80	0.71	0.74
	Coefficient of determination R^2^ (-)	0.99	0.99	0.99	0.99
Area of the hysteresis loop	∆P (Pa/s)	14,104.28	11,393.72	18,218.56	20,841.57

YC—yoghurt produced from untreated reconstituted milk; YHP—yoghurt produced from HP-treated reconstituted milk; YC1—yoghurt produced from untreated reconstituted milk after storage; YHP1—yoghurt produced from HP-treated reconstituted milk after storage.

**Table 5:** Textural properties of yoghurts.

Textural Properties	Yoghurt			
	YC	YHP	YC1	YHP1
WHC (%)	30.83 ^a^ ± 0.89	32.51 ^b^ ± 1.64	34.74 ^c^ ± 0.09	36.37 ^d^ ± 0.26
Firmness (N)	0.74 ^a^ ± 0.12	0.97 ^b^ ± 0.13	0.73 ^a^ ± 0.04	1.04 ^b^ ± 0.11
Consistency (N s)	18.53 ^a^ ± 1.83	24.94 ^b^ ± 2.74	17.54 ^a^ ± 0.23	26.86 ^b^ ± 2.04
Cohesiveness (N)	−0.82 ^a^ ± 0.22	−1.13 ^b^ ± 0.04	−0.62 ^a^ ± 0.07	−0.83 ^a^ ± 0.14

Means ± standard deviation (*n* = 3); ^a, b, c, d^—mean values marked with different letters in the same row differ at *p* ≤ 0.05; YC—yoghurt produced from untreated reconstituted milk; YHP—yoghurt produced from HP-treated reconstituted milk; YC1—yoghurt produced from untreated reconstituted milk after storage; YHP1—yoghurt produced from HP-treated reconstituted milk after storage.

**Table 6:** Mean values of the sensory attributes of caprine milk yoghurts.

Sensory Attributes	Yoghurt				*p*-Value
	YC	YHP	YC1	YHP1	
Appearance					
Color uniformity	6.6	6.5	6.6	6.8	>0.05
Creamy color	5.3	5.0	5.4	5.8	>0.05
Whey syneresis	1.3	1.1	1.5	1.5	>0.05
Aroma					
Yoghurt-like	5.0 ^b^	4.3 ^a^	5.5 ^b^	5.5 ^b^	0.025
Sour	2.8 ^a^	2.5 ^a^	3.3 ^b^	3.5 ^b^	0.048
Goaty	2.0	1.9	2.5	1.9	>0.05
Atypical	1.0	1.0	1.0	1.0	>0.05
Consistency					
Uniform	7.0	7.0	6.8	6.6	>0.05
Thick	2.8	3.4	3.1	3.6	>0.05
Ropy	3.9 ^d^	3.5 ^c^	2.6 ^b^	1.5 ^a^	0.000
Gelatinous	4.0 ^a^	5.6 ^b^	4.6 ^a^	5.8 ^b^	0.009
Smooth mouthfeel	6.9	6.9	6.9	6.8	>0.05
Taste					
Yoghurt-like	4.6 ^a^	5.0 ^a^	5.3 ^ab^	5.6 ^b^	0.035
Sour	4.1	4.0	4.8	4.1	>0.05
Bitter	1.0	1.0	1.0	1.0	>0.05
Sweet	2.9	2.9	2.5	2.6	>0.05
Salty	1.0	1.0	1.0	1.0	>0.05
Goaty	3.1	3.1	3.5	3.0	>0.05
Foreign	1.0	1.0	1.0	1.0	>0.05
Overall Acceptability	5.1 ^ab^	5.6 ^b^	4.5 ^a^	5.8 ^b^	0.045

^a, b, c, d^—mean values (n = 8) marked with different letters in rows differ at *p* ≤ 0.05; YC—yoghurt produced from untreated reconstituted milk; YHP—yoghurt produced from HP-treated reconstituted milk; YC1—yoghurt produced from untreated reconstituted milk after storage; YHP1—yoghurt produced from HP-treated reconstituted milk after storage.

## Data Availability

The original contributions presented in the study are included in the article, further inquiries can be directed to the corresponding author.
